# Editorial: The role of platelet derivatives in regenerative medicine

**DOI:** 10.3389/fcell.2026.1887498

**Published:** 2026-06-04

**Authors:** Eleonora Stanca, Fabrizio Damiano, Andrea Palermo

**Affiliations:** Department of Experimental Medicine, University of Salento, Lecce, Italy

**Keywords:** platelet derivative, platelet lysate, platelet-rich fibrin (PRF), platelet-rich plasma (PRP), regenerative medicine

Over the last decade, autologous platelet-derived products have progressively emerged as central tools in regenerative medicine, finding applications across remarkably diverse clinical and experimental settings. The growing body of evidence suggests that the regenerative potential of platelet derivatives extends far beyond a simple reservoir of growth factors. Rather, these biological products appear to function as dynamic and context-dependent regulators of tissue repair. The studies present in this Research Topic emphasize how platelet-rich plasma (PRP), platelet-rich fibrin (PRF), concentrated growth factors, and human platelet lysate (HPL) can modulate fundamental cellular programs involved in regeneration, including proliferation, migration, survival, differentiation, extracellular matrix remodeling, and immunomodulation. Importantly, these effects are observed in tissues characterized by profoundly different biological architectures and regenerative capacities, such as bone, cartilage, endometrium, and temporomandibular joint fibrocartilage. This apparent biological versatility raises an important mechanistic consideration: the efficacy of platelet derivatives likely resides not in the action of a single mediator, but in the simultaneous presence of multiple bioactive molecules capable of orchestrating tissue-specific signaling responses.

Indeed, platelet derivatives contain a complex mixture of growth factors, cytokines, chemokines, adhesive proteins, extracellular vesicles, and fibrin architectures that collectively shape the local microenvironment.

This topic highlight the activation of signaling pathways such as MAPK/ERK, PI3K/AKT, TGF-β-related cascades, and PDGF-dependent pathways (Mourao and Alves; Zhao et al.), which regulate stromal cell activation, stem cell fate, inflammatory balance, and matrix synthesis. The ability of these mediators to engage membrane receptors expressed by different resident cell populations may explain why platelet derivatives can promote regenerative responses in highly heterogeneous tissues. In this view, platelet concentrates may be interpreted as biological “adaptive systems,” capable of interacting with local cellular niches and amplifying endogenous repair programs according to tissue-specific requirements.

An additional emerging concept concerns the temporal dimension of growth factor delivery. Comparative studies between PRP and PRF demonstrate that not only the molecular composition, but also the kinetics of release significantly influence biological outcomes. PRF, for example, appears to provide a more sustained release of growth factors, prolonging anti-apoptotic and pro-migratory effects in endometrial stromal cells and potentially offering longer-lasting regenerative stimulation (Yuan et al.). Similarly, fibrin organization and scaffold-like properties may influence cell recruitment and spatial signaling gradients within damaged tissues.

The translational potential of platelet derivatives is further reinforced by advances in manufacturing technologies. Wendland and colleagues demonstrated that lyophilized human platelet lysate maintains growth factor content and mesenchymal stromal cell-supporting properties comparable to fresh preparations, while offering improved storage stability and broader applicability. These findings are particularly relevant for the future clinical standardization and large-scale implementation of platelet-derived biologics. Lyophilization may represent a crucial step toward overcoming some of the major limitations historically associated with platelet products, including batch variability and limited shelf life (Wendland et al.).

At the clinical level, encouraging results continue to emerge in challenging regenerative contexts. Studies on tibial and femoral nonunion suggest that platelet derivatives can significantly enhance bone healing when combined with biomechanical stabilization and grafting procedures. Notably, these approaches appear particularly valuable in refractory conditions characterized by impaired healing capacity and repeated surgical failure (Lu et al.; Chen et al.). Similarly, applications in temporomandibular disorders and cartilage regeneration underscore the capacity of platelet-derived products to support stem cell recruitment, chondrogenic differentiation, and inflammation resolution in tissues with intrinsically poor vascularization and limited regenerative potential (Lin et al.; Zhao et al.).

Despite these promising observations, one of the major challenges in the field remains the profound heterogeneity of platelet-derived formulations. Variability in centrifugation protocols, leukocyte content, anticoagulants, activation methods, donor-related factors, and fibrin organization continues to complicate comparisons between studies and contributes to inconsistent clinical outcomes. As highlighted by several reviews included in this Research Topic, the field is now moving toward more rigorous frameworks for standardization, including classification systems, minimum reporting guidelines, and quantitative approaches such as DEPA-based characterization. These efforts are essential not only to improve reproducibility, but also to establish meaningful correlations between biological composition, mechanistic activity, and clinical efficacy.

Collectively, the studies gathered in this Research Topic reinforce the concept that platelet derivatives should not be considered merely supportive adjuncts, but rather multifunctional biological platforms capable of modulating regenerative processes through integrated signaling networks. Their broad applicability across tissues suggests that regenerative efficacy may emerge from the interaction between a conserved pool of platelet-derived mediators and tissue-specific cellular responses.

